# Neuroinflammation mediated by IL-1β increases susceptibility of dopamine neurons to degeneration in an animal model of Parkinson's disease

**DOI:** 10.1186/1742-2094-5-8

**Published:** 2008-02-27

**Authors:** James B Koprich, Casper Reske-Nielsen, Prabhakar Mithal, Ole Isacson

**Affiliations:** 1 Center for Neuroregeneration Research, Harvard Medical School/McLean Hospital, Belmont, MA, 02478, USA; 2 Udall Parkinson's Disease Research Center of Excellence, Harvard Medical School/McLean Hospital, Belmont, MA, 02478, USA; 3 Program in Neuroscience, Harvard Medical School, Boston, MA, 02115, USA

## Abstract

**Background:**

The etiology of Parkinson's disease (PD) remains elusive despite identification of several genetic mutations. It is more likely that multiple factors converge to give rise to PD than any single cause. Here we report that inflammation can trigger degeneration of dopamine (DA) neurons in an animal model of Parkinson's disease.

**Methods:**

We examined the effects of inflammation on the progressive 6-OHDA rat model of Parkinson's disease using immunohistochemistry, multiplex ELISA, and cell counting stereology.

**Results:**

We show that a non-toxic dose of lipopolysaccharide (LPS) induced secretion of cytokines and predisposed DA neurons to be more vulnerable to a subsequent low dose of 6-hydroxydopamine. Alterations in cytokines, prominently an increase in interleukin-1beta (IL-1β), were identified as being potential mediators of this effect that was associated with activation of microglia. Administration of an interleukin-1 receptor antagonist resulted in significant reductions in tumor necrosis factor-α and interferon-γ and attenuated the augmented loss of DA neurons caused by the LPS-induced sensitization to dopaminergic degeneration.

**Conclusion:**

These data provide insight into the etiology of PD and support a role for inflammation as a risk factor for the development of neurodegenerative disease.

## Background

Parkinson's disease (PD), to a large extent, is precipitated by unknown etiological factors. Classically, PD has been associated with multiple causes ranging from post-encephalitic PD [1,2] to hereditary PD [3-7] in which genetic mutations have been associated such as, α-synuclein [5], parkin [4], DJ-1 [3], PINK1 [6], and LRRK2 [7]. Genetics, however unlikely acts alone in typical PD. Using a large cohort of twins Tanner et al. demonstrated no overall difference in concordance for PD between monozygotic and dizygotic pairs especially when disease onset was greater than 50 years [8]. Gaining attention is the concept that idiopathic PD results from combinations of multiple risk factors that include, age, genetic predisposition, environmental toxins, and possibly inflammation [9,10]. The central tenet being that each successive risk factor in turn engages compensatory mechanisms and eventually compromises neuronal health beyond recovery [11].

In several epidemiological reports, non-steroidal anti-inflammatory drugs (NSAIDs) were associated with a reduced risk of developing PD [12-14]. However, in two other investigations, although using significantly smaller sample sizes, no such association was found [15,16]. Chen et al reported that men with elevated plasma levels of interleukin-6 have an increased risk of developing PD [17]. Influenza [18,19], traumatic brain injury [20,21] and allergic rhinitis [22] are speculated as being risk factors for PD, although these have yet to be verified.

Evidence of an enduring neuroinflammation examined well into the course of PD has, however, been documented. In the substantia nigra (SN), inflammation is represented by activated microglia with increased expression of major histocompatibility complex-II [23] and elevated levels of pro-inflammatory cytokines including, tumor necrosis factor-α (TNF-α) [24,25], interleukin-1β (IL-1β) [25], and interferon-γ [25]. Using magnetic resonance spectroscopy (MRS), we have shown, in the striatum of 1-methyl 4-phenyl 1,2,3,6-tetrahydropyridine (MPTP) treated primates and PD patients, a large increase in the choline/creatine ratio, possibly reflective of gliosis or macrophage activity [26]. In addition, intercellular adhesion molecule-1 (ICAM-1, CD54) is highly expressed on astrocytes in the SN of PD patients and accordingly its receptor, lymphocyte function-associated antigen-1 (LFA-1) was observed on microglia and infiltrating leukocytes [27], indicating a role for immune regulation outside of the CNS.

Several investigators suggest that treatments aimed at controlling inflammation may be beneficial in curbing cell loss associated with PD progression. For example, cyclooxygenase-2 inhibition has been shown to reduce cell loss in rodent [28] and non-human primate models of PD (Sánchez Pernaute R, Brownell AL, Viñuela A, Zhu A, Koprich JB, McDowell J, Pagel O'Malley J, Wang X, Yu M, Isacson O, submitted). In addition, minocycline [29], dexamethasone [30], blocking tumor necrosis factor alpha (TNF-α) [31] and nNOS (neuronal nitric oxide synthase) inhibition [32] have all reduced inflammation and demonstrated efficacy in animal models of PD. While these data suggest that inflammation is involved in PD progression, less is known about its role in disease onset.

The intra-striatal 6-OHDA rat model of PD produces an irreversible and progressive loss in DA neurons of the SN [33]. Interestingly this lesion results in an inflammatory response [34] that can only be controlled approximately 12 days after lesion onset [28], making this an ideal animal model for gaining insights into inflammation and PD pathogenesis. With some modifications, we utilized this animal model to examine whether neuroinflammation contributes to the onset of PD. To this end we induced a non-toxic inflammation in the SN using a low dose of lipopolysaccharide (LPS) followed by a low dose of 6-OHDA to simulate the early stages of PD. We report that preexisting inflammation increases the magnitude of cell loss produced by 6-OHDA. Furthermore, we identified IL-1β as a potential mediator of this effect and were able to reverse the cell loss in our paradigm through administration of an interleukin-1 receptor antagonist.

## Methods

### Animals

Female Sprague-Dawley rats weighing ~280 g (Charles River Laboratories) were used in all animal experiments. All rat studies were approved by the McLean Hospital Institutional Animal Care and Use Committee.

### Stereotaxic surgery

All stereotaxic coordinates were derived from the Rats Atlas of Paxinos and Watson [35]. For each surgery animals were deeply anesthetized with ketamine and xylazine (60 mg/kg and 3 mg/kg respectively, i.m.). *Substantia nigra LPS injection*. Animals received a single 2.0 μl stereotaxic injection of either lipopolysaccharide (0.09 μg LPS, serotype 026:B6, Sigma-Aldrich) or saline and delivered at a rate of 0.5 μl/min using microinfusion pumps (Stoelting Co, Wood Dale, IL) with a 5 min wait time after injection. SN injection coordinates were as follows: AP -4.8, ML -2.0, DV -7.3, and tooth bar set at -3.3. *6-OHDA intra-striatal injection*. Animals received a single 3.5 μl stereotaxic injection of 6-OHDA (total dose = 5.0 or 22.5 μg 6-OHDA prepared as free base, Sigma-Aldrich) delivered at a rate of 0.5 μl/min and a 5 min wait time after injection. Striatum injection coordinates were as follows: AP +0.2, ML -3.0, DV -5.0, and tooth bar set at -3.3. The lesion was allowed to progress for 3 weeks after which animals were sacrificed for post mortem analyses.

### Interleukin-1ra (IL-1ra) administration

IL-1ra (Anakinra) (Amgen, Thousand Oaks, CA) was purchased through McLean Hospital Pharmacy and prepared as a 100 mg/mL solution. Osmotic pumps (model 2ML1, Alzet, Cupertino, CA) were filled with either IL-1ra (to deliver: 3.64 mg/kg/hr IL-1ra, s.c.) or vehicle and implanted subcutaneously under ketamine and xylazine anesthesia (60 mg/kg and 3 mg/kg respectively, i.m.). Pumps were replaced weekly until completion of the study. IL-1ra has previously been shown to cross the blood brain barrier and achieve therapeutic concentrations [36,37].

### Perfusions and tissue handling

Animals were deeply anesthetized with an i.p. injection of sodium pentobarbital and were sacrificed by exsanguination with the aid of ice-cold saline perfusion. For immunohistochemistry, the brains were then fixed with a 4% paraformaldehyde solution. The brains were then removed from the skull and placed in fresh 4% paraformaldehyde solution for 1 h, and equilibrated through 20% and 30% sucrose solutions and refrigerated until cutting for immunohistochemistry. For ELISA, brains were rapidly removed after saline perfusion and sliced coronally using a tissue chopper set to 1 mm (Campden Instruments Ltd., Lafayette, IN). On an inverted glass Petri dish over ice, regions of interest (striatum and substantia nigra region) were dissected from the individual 1 mm tissue slices, frozen on dry ice, and stored at -80°C.

### Immunohistochemistry

Brains were cut frozen in the coronal plane at a thickness of 40 μm on a sliding microtome and six series of sections were stored in cryoprotectant. One-sixth series of sections were processed for visualization of tyrosine hydroxylase (TH) or CD11b via the biotin-labeled antibody procedure. Briefly, following several washes in a PBS solution containing 0.01% Triton ×-100 (PBS-T), endogenous peroxidase was quenched in a 3% hydrogen peroxide solution and background staining was then inhibited in a 5% normal goat serum solution. Tissue was then incubated with rabbit anti-TH antibody overnight (1:1000, Pel-Freez, Rogers, AR) or mouse anti-CDllb (1:100, Serotec, UK). After three washes in PBS-T, sections were sequentially incubated in biotinylated goat antibodies IgG (1:500; Vector, Burlingame, CA) for 1 h and the Elite avidin-biotin complex (ABC Kits; Vector, Burlingame, CA) for 1 h separated by three washes in PBS. TH and CD11b immuno staining was visualized following a reaction with 3,3-diaminobenzidine (Vector). Sections were then mounted on glass slides, allowed to dry, dipped into dH_2_0, dehydrated through graded alcohol (70%, 95%, 100%), cleared in xylenes, and coverslipped with DPX mounting medium.

### Multiplex ELISA

Tissue samples were collected and suspended in lysis buffer (TPER, Peirce, Rockford, IL). In addition, phosphatase inhibitors I-II (1:100) and protease inhibitors (1:100) were added fresh prior to cell lysis (Sigma-Aldrich, P2850, P5276, and P8340 respectively). Following cell lysis, the homogenate was centrifuged, a portion of the supernatant was reserved for protein determination (BCA Assay, Pierce, Rockford, IL) and the remaining was stored at -20°C. Samples were analyzed for the simultaneous detection of IL-1β, IL-2, IL-4, IL-6, IL-10, IFNγ, TNF-α, MCP-1, and Fractalkine using a multiplex ELISA based format and performed in duplicate. Testing was performed independently through the Searchlight Testing Service (Pierce, Thermo Fisher Scientific, Woburn, MA).

### Cell counting

Estimates of TH-positive neuronal number within the substantia nigra (SN) were performed using Stereo Investigator software (MBF Bioscience, Williston, VT) and stereologic principles. Six sections, each separated by 240 μm from the anterior to the posterior SN, were used for counting of each case. Stereology was performed using a Zeiss Axiovert microscope (Zeiss, Thornwood, NY) coupled to an Optronics Microfire digital camera (Goleta, CA) for visualization of tissue sections. The total number of TH-positive neurons was estimated from coded slides using the optical fractionator method. For each tissue section analyzed, section thickness was assessed empirically and guard zones of 2 μm thickness were used at the top and bottom of each section. The SN was outlined under low magnification (2.5×) and approximately 54% of the outlined region was analyzed using a systematic random sampling design generated with the following stereologic parameters: grid size, 170 × 170 μm; counting frame size, 125 × 125 μm; and dissector height, 18 μm. Neurons were counted under 40× magnification. The coefficients of error (CE) were calculated according to the procedure of West and colleagues, values <0.10 were accepted [38].

### Statistical analyses

1-way ANOVA was used to compare more than 2 groups on the same dependent measure and post-hoc analysis (Holm-Sidak) was conducted to reveal simple effects. Student t-tests were used to compare two groups on a single dependent measure. All analyses were conducted using Sigma Stat v. 3.0 (San Jose, CA). All tests were considered significant at p < 0.05.

## Results

### Preexisting neuroinflammation contributes to 6-OHDA induced dopamine neuron loss in the substantia nigra

**Figure 1:**
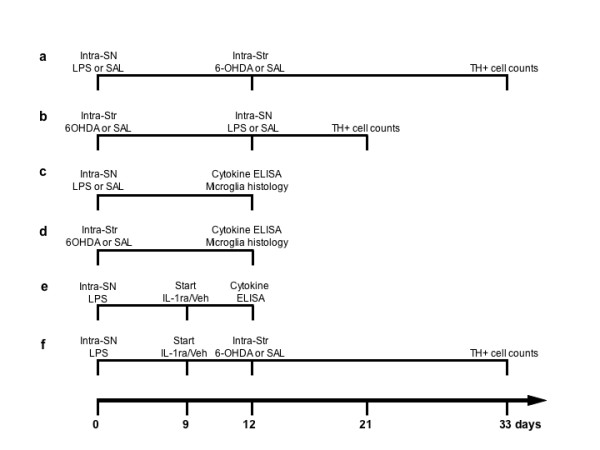
Experimental timelines. Each timeline represents in vivo experimental procedures and postmortem analyses conducted. Number of animals in each group: (*A*) n = 6, (*B*) n = 6, (*C*) n = 7, (*D*) n = 7, (*E*) n = 5, (*F*) n = 8. LPS, lipopolysaccharide; 6-OHDA, 6-hydroxydopamine, SAL, saline; IL-1ra, interluekin-1 receptor antagonist; veh, vehicle; SN, substantia nigra, Str, striatum.

**Figure 2:**
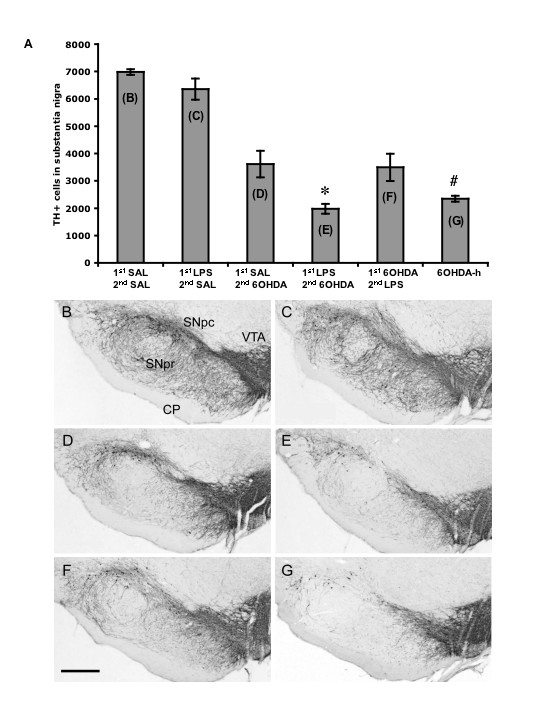
*(A) *Tyrosine hydroxylase positive (TH+) cell counts in the substantia nigra (SN). *(B) *Animals received, into the SN, saline (SAL) and 12 days later received an intra-striatal injection of SAL. *(C) *Animals received lipopolysaccharide (LPS) into the SN and 12 days later received an intra-striatal injection of SAL. *(D) *Animals received SAL into the SN and 12 days later received an intra-striatal injection of 6-OHDA (5.0 μg). *(E) *Animals received LPS into the SN and 12 days later received an intra-striatal injection of 6-OHDA (5.0 μg). *(F) *Animals received an intra-striatal injection of 6-OHDA (5.0 μg) and 12 days later received LPS into the SN. *(G) *Animals received an intra-striatal injection of 6-OHDA-h (high) (22.5 μg). Letters inside bars of panel *A *represent respective images in panels below. 21 days was allowed following 6-OHDA injection in all conditions. There was an overall main effect across groups (ANOVA, *p *< 0.001). LPS injection into the SN was non-toxic to TH+ neurons (*B vs. C*, *NS*). LPS injection prior to 6-OHDA administration increased the amount of TH+ cell loss compared to SAL prior to 6-OHDA (*D vs. E*, *p < 0.05, post-hoc Holm-Sidak). Intra-striatal injection of 6-OHDA followed by injection of LPS in the SN produced no greater cell loss (*D vs. F*, *NS*). The high dose of 6-OHDA produced cell loss that was not significantly different from a low dose of 6-OHDA with prior exposure to LPS (*E vs. G, NS*), however the high dose of 6-OHDA was significantly greater than other conditions receiving the lower dose of 6-OHDA (*G vs. D and F*, # *p *< 0.05, post-hoc Holm-Sidak). 1^st^, first injection; 2^nd^, second injection; SNpc, pars compacta; SNpr, pars reticulata; VTA, ventral tegmental area; CP, cerebral peduncle; scale bar, 1.0 mm; magnification, 2.5×. Error bars, ± SEM; n = 6 per condition.

Rats received a single intra-nigral injection of LPS (0.09 μg) either prior to or following an intra-striatal injection of 6-OHDA. Each condition was allowed 21 days post 6-OHDA administrations for consistent comparison of lesion severity (Fig. 1a–b). This experiment was designed to test the hypothesis of whether direct inflammation in the midbrain contributes to the onset and/or the progression of SN DA neuron cell loss. One-way ANOVA revealed an overall main effect indicating significant differences in cell counts across all treatment conditions [F(5,33) = 42.11, p < 0.001]. More specifically through post-hoc analysis, LPS injection into the SN followed by saline injection into the striatum produced no significant TH-ir cell loss in the SN compared to saline injections alone (t = 1.30, *NS*) (Fig. 2b–c), indicating that the dose of LPS chosen did not produce cell loss on its own. This dose of LPS was selected based on a dose response study determining the presence of an inflammatory response without overt TH-ir cell loss (unpublished data). Animals receiving an injection of saline into the SN followed by intra-striatal injection of 6-OHDA (5.0 μg) experienced a 52% loss of TH-ir cells in the SN (t = 11.62, p < 0.001) compared to control (Fig. 2b vs. 2d). Prior neuroinflammation generated by LPS followed by intra-striatal injection of 6-OHDA produced a greater TH-ir cell loss (72%) compared to intra-nigral injection of saline followed by intra-striatal injection of 6-OHDA (t = 3.95, p < 0.05) (Fig. 2d–e). In contrast, LPS injection into the SN during an ongoing 6-OHDA induced degeneration produced no further cell loss as compared to 6-OHDA in combination with saline (t = 0.36, *NS*) (Fig. 2d vs. 2f).

To provide context to the combined lesion effect of LPS followed by 6-OHDA (5.0 μg) we also included a group that received a higher dose of 6-OHDA (22.5 μg) alone. The amount of cell loss produced by this dose (66%) was not significantly different from animals receiving LPS followed by 5.0 μg of 6-OHDA (t = 0.84, *NS*) (Fig. 2g vs. 2e).

### Microglia activation in the ventral midbrain following administration of LPS and 6-OHDA

**Figure 3:**
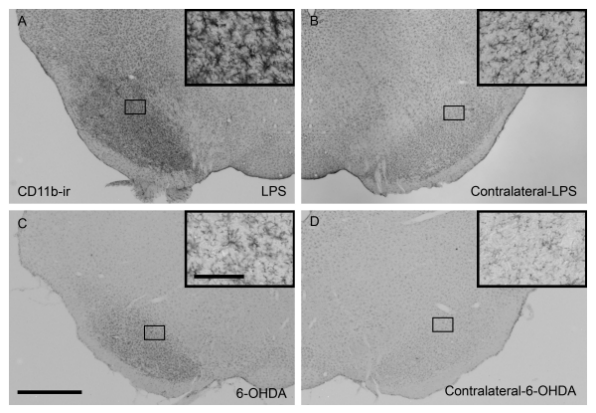
Representative coronal sections of microglia in the ventral midbrain at the level of the substantia nigra (SN) visualized by CD11b-immunoreactivity (-ir). (*A*) activated microglia in the SN of a rat injected in the SN with LPS 12 days prior and (*B*) the contralateral side. (*C*) low level of microglia activation 12 days following an intra-striatal injection of 6-OHDA (5.0 μg) and (*D*) the contralateral side showing resting microglia. Insets are 20× images taken from the area outlined in lower magnification pictures. Scale bar of inset, 100 μm; scale bar of low magnification images (2.5×), 1.0 mm).

In the experiment described above, LPS administration increased the amount of cell loss produced by 6-OHDA. We next wanted to understand how LPS produced increased vulnerability of SN DA neurons in this paradigm. In these experiments, animals were killed 12 days following either injection of LPS (intra-nigral) or 6-OHDA (intra-striatal) (Fig. 1c–d). We next determined the degree of microglia activation using CD11b as a marker. Greater staining intensity and a shift in cell morphology towards full activation (resting microglia appear as having smaller soma with fine processes, compared to activated microglia having larger soma and short thicker processes) were used as the basis of comparison [39]. Animals that received LPS or 6-OHDA had greater staining intensity of CD11b and a more advanced activated morphology compared to their respective contralateral sides (Fig. 3a–d). Furthermore, LPS induced changes appeared to be greater than that produced by 6-OHDA (Fig. 3a vs. 3c). These data served to indicate that the dose of LPS injected into the SN produced inflammation in the absence of overt TH+ cell loss (Fig. 2c).

### Cytokine and chemokine alterations following LPS injection into the substantia nigra

We next screened a panel of cytokines and chemokines in order to obtain a more in depth analysis of the increased susceptibility of DA neurons produced by the bacterial endotoxin, LPS. A new set of animals was injected, as before, with LPS, 6-OHDA, or saline as respective controls (Fig. 1d–e). A panel of cytokines and chemokines was selected and quantified in the SN and striatum of each animal using a multiplex ELISA format. Table 1 shows the values obtained from SN tissue alone following either LPS/saline or 6-OHDA/saline injections. We found that twelve days following LPS injection into the SN there was a significant increase in SN IL-1β [t(11) = 6.25, p < 0.001] and MCP-1 [t(11) = 2.57, p < 0.05) and a significant decrease in IL-4 [t(11) = 3.15, p < 0.01] compared to saline controls. No significant changes were seen in the SN following a 6-OHDA injection (Table 1). We also quantified levels in the striatum following LPS/saline or 6-OHDA/saline injections and found no significant differences in protein levels in either condition (Table 2).

**Table 1:** Substantia nigra tissue ELISA. Levels of cytokines/chemokines in the substantia nigra 12 days following an intra-nigra injection of LPS/saline or intra-striatal injection of 6-OHDA/saline.

**Cytokine or Chemokine**	**Substantia nigra protein levels (pg/mg) ± SEM**			
	
	**Substantia nigra**		**Striatum**	
	**SAL**	**LPS**	**SAL**	**6OHDA**
**IL-1β**	7.17 ± 0.64	13.54 ± 0.69**	7.58 ± 0.64	6.92 ± 0.71
**MCP-1**	5.12 ± 0.71	8.14 ± 0.64*	3.96 ± 0.25	5.07 ± 0.42
**IL-4**	0.46 ± 0.07	0.26 ± 0.01*	0.63 ± 0.16	0.45 ± 0.03
**IFN-γ**	9.88 ± 1.48	8.02 ± 0.49	13.00 ± 1.32	10.11 ± 1.13
**IL-10**	1.73 ± 0.05	1.46 ± 0.17	1.74 ± 0.13	1.61 ± 0.10
**IL-2**	20.58 ± 0.98	23.17 ± 4.33	24.05 ± 1.71	22.84 ± 1.29
**TNF-α**	5.66 ± 0.20	6.82 ± 0.18	6.92 ± 0.80	6.47 ± 0.43
**Fractalkine**	20.67 ± 2.07	18.40 ± 1.20	19.91 ± 1.04	20.73 ± 0.55
**IL-6**	5.95 ± 0.60	5.32 ± 0.37	5.65 ± 0.49	5.97 ± 0.40

*p < 0.05; **p < 0.01; SAL, saline; LPS, lipopolysaccharide

**Table 2:** Striatum tissue ELISA. Levels of cytokines/chemokines in the striatum12 days following an intra-nigral injection of LPS/saline or intra-striatal injection of 6-OHDA/saline.

**Cytokine or Chemokine**	**Striatum protein levels (pg/mg) ± SEM**			
	
	**Substantia nigra**		**Striatum**	
	**SAL**	**LPS**	**SAL**	**6OHDA**
**IL-1β**	2.29 ± 0.45	2.41 ± 0.26	3.03 ± 0.48	2.96 ± 0.35
**MCP-1**	0.76 ± 0.06	0.79 ± 0.06	6.20 ± 1.24	4.36 ± 1.76
**IL-4**	0.20 ± 0.03	0.25 ± 0.05	0.13 ± 0.02	0.17 ± 0.04
**IFN-γ**	3.58 ± 0.46	3.76 ± 0.39	3.95 ± 0.41	3.89 ± 0.26
**IL-10**	0.61 ± 0.07	0.57 ± 0.04	0.69 ± 0.04	0.68 ± 0.05
**IL-2**	10.02 ± 0.70	10.01 ± 0.79	10.08 ± 0.36	10.99 ± 0.92
**TNF-α**	2.50 ± 0.20	2.44 ± 0.18	2.43 ± 0.27	2.56 ± 0.14
**Fractalkine**	14.33 ± 0.80	13.97 ± 0.21	15.54 ± 0.71	16.59 ± 0.86
**IL-6**	2.81 ± 0.27	3.19 ± 0.24	3.14 ± 0.46	2.78 ± 0.29

SAL, saline; LPS, lipopolysaccharide

### Administration of IL-1ra produces changes in ventral midbrain cytokine content and reduces the cell loss produced by LPS and 6-OHDA

From the cytokine/chemokine profile generated in the previous experiment we selected IL-1β as being potentially involved with the increased 6-OHDA degeneration induced by prior LPS. To test this hypothesis we first administered an IL-1ra or vehicle to the periphery via subcutaneous osmotic pumps (3.64 mg/kg/hr IL-1ra, s.c.) to animals 9 days following a LPS injection into the SN. On the twelfth day animals were sacrificed and processed for ventral midbrain cytokine/chemokine levels (Fig. 1e). We found that LPS exposed animals treated with IL-1ra had a significant decrease in TNF-α [t(8) = 3.28, p < 0.01] and IFN-γ [t(8) = 2.47, p < 0.05] compared to LPS exposed animals receiving vehicle (Table 3). No significant changes in IL-1β were observed, however changes in TNF-α and IFN-γ are indicative of alterations in IL-1 signaling [40]. These data demonstrated that peripheral IL-1ra administration can exert its effects across the blood brain barrier.

**Table 3:** Cytokine and chemokine protein levels (pg/mg) ± SEM in substantia nigra of animals treated with IL-1ra or vehicle. All animals received an intra-nigra injection of LPS 9 days prior to drug treatment. IL-1ra treatment lasted for 3 days.

**Cytokine/Chemokine**	**Vehicle**	**IL-1ra**
**IL-1β**	9.32 ± 1.38	9.61 ± 2.48
**MCP-1**	12.48 ± 2.58	10.56 ± 1.71
**IFN-γ**	2.42 ± 0.38	1.32 ± 0.23*
**IL-10**	0.22 ± 0.03	0.15 ± 0.02
**IL-1α**	1.27 ± 0.04	1.03 ± 0.17
**TNF-α**	2.35 ± 0.29	1.16 ± 0.22*
**Fractalkine**	14.64 ± 1.37	15.21 ± 1.45
**IL-6**	13.18 ± 2.71	9.13 ± 1.25

*p < 0.05; IL-4 was below detectable limits.

**Figure 4:**
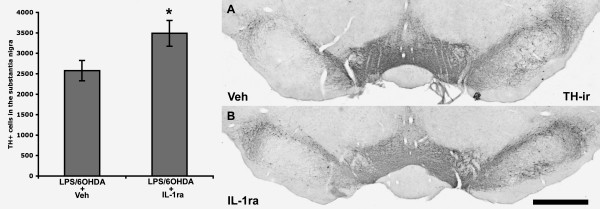
Interleukin-1 receptor antagonist (IL-1ra) reduces the amount of tyrosine hydroxylase-immunoreactive (TH-ir) cell loss associated with LPS and 6-OHDA (t-test, *p < 0.05). Each animal received LPS into the substantia nigra (SN), 9 days later started on either IL-1ra or vehicle (Veh) which continued through the experiment, and all animals then received an intra-striatal injection of 6-OHDA (5.0 μg) and were allowed 21 days until post-mortem analyses. Magnification, 2.5×; scale bar, 1.0 mm. Error bars, ± SEM; n = 8 per condition.

We next conducted a study to test whether IL-1ra administration could prevent the synergistic increase in TH-ir cell loss produced by LPS and 6-OHDA. All animals received an intra-nigral injection of LPS, followed by either IL-1ra or vehicle from day 9 until sacrifice, and finally, all animals received an intra-striatal injection of 6-OHDA (Fig. 1f). Each animal receiving IL-1ra had significantly greater IL-1ra serum protein levels, measured by ELISA, at the end of the study (data not shown). Postmortem analysis revealed that significantly more TH-ir cells remained in the SN of animals that received IL-1ra compared to vehicle treated controls [t(11) = 2.54, p < 0.05] (Fig. 4), indicating that blocking of IL-1 signaling significantly reduces the degree of degeneration produced by the combination of LPS and 6-OHDA.

## Discussion

The present study demonstrates that preexisting inflammation in the brain, produced locally by LPS, potentiates the amount of DA neuron loss generated in the intra-striatal 6-OHDA rat model of PD. Furthermore, we report that LPS elevates IL-1β levels in the midbrain and is important in creating this increase in vulnerability. This was evidenced by attenuated cell loss after administration of an IL-1 receptor antagonist. These data support a role for inflammation as a potential component in the development of idiopathic PD.

LPS is derived from gram-negative endotoxin bacterium that acts through a membrane bound receptor complex found on the surface of microglia [41]. Its primary action initiates nuclear translocation of transcription factor nuclear factor κB (NF-κB) and the upregulation of pro-inflammatory gene expression [42]. Following injection into the midbrain, LPS has been shown to activate microglia [43], increase pro-inflammatory cytokine levels [44], and directly result in death of DA neurons [45-50] that is dependent on tyrosine hydroxylase activity [47], while sparing other phenotypes (Gamma-aminobutyric acid and serotonin neurons) [48]. Direct injection of LPS into other brain areas (hippocampus and cortex) also produces no overt cell loss [51], indicating that DA neurons possess a relative vulnerability to the direct effects of inflammation.

In the current study we found that a non-DA-toxic dose of LPS (0.09 μg) delivered to the SN produces activation of microglia and alterations in select cytokines as measured twelve days later. In the SN, the pro-inflammatory cytokine IL-1β and the chemokine, MCP-1 were significantly elevated, while there was a significant decrease in the anti-inflammatory cytokine, IL-4. IL-1β is a potent pro-inflammatory cytokine that acts through IL-1 receptors found on numerous cell types including neurons and microglia. IL-1 signaling leads to NF-κB mediated expression of pro-inflammatory cytokines, thereby perpetuating the cycle of inflammation initiated by LPS. In PD patients, IL-1β is elevated in CSF [52], striatum [53], and SN [25]. Genetic studies have revealed that the T allele in the regulatory region of the IL-1β gene (-511) is elevated in PD patients and may be responsible for increased IL-1β expression [54,55]. In relation to the current study, overexpresssion of IL-1β in these patients may have lead to increased DA neuron vulnerability to exogenous toxins and an increased risk for PD onset. In contrast, it has been shown that PD patients of a Japanese cohort with higher expression of IL-β had a later age of disease onset compared to lower expressing patients [56]. These data indicate that IL-1β may be important in PD onset and that more research needs to be done to determine its exact role. In rat, it has been shown that direct delivery of IL-1β to the midbrain produces DA neuron degeneration in the SN [57]. The combination of independently non-toxic doses of quinolinic acid and IL-1β has been shown to synergistically produce pyramidal cell loss in the hippocampus [58]. Interleukin-1β is therefore a potent regulator of inflammation and has been shown to be associated with neurodegenerative disease.

A sustained inflammatory response to our single LPS injection is evidenced by increases in MCP-1 and a stable state of microglia activation. MCP-1 is expressed and released by DA neurons when an activating stimulus continues to be present [39] and through interaction with its microglial receptor, chemokine receptor 2 (CCR2) [59,60], is able to direct the chemotaxis of microglia towards neurons to promote cell-cell contact [39]. Persistent levels of IL-1β, MCP-1 and activated microglia in our paradigm (at least 12 days following LPS injection) may, at least in part, be responsible for the increase in SN DA neuron susceptibility to 6-OHDA we observed in the present study.

To provide context to our combined insult paradigm, in terms of 6-OHDA lesion severity, we also included a group that received a higher dose of 6-OHDA alone. We found that LPS delivered to the SN prior to the intra-striatal injection of 6-OHDA (5.0 μg) produced the same degree of TH+ cell loss in the SN as a striatal injection of 22.5 μg of 6-OHDA (*p *> 0.05, *NS*). These data indicate that preexisting neuroinflammation potentiates the effects of a 5.0 μg 6-OHDA to that of a 22.5 μg dose.

Through systemic administration of IL-1ra we were able to reverse the vulnerability produced by LPS and therefore eliminate its contribution to 6-OHDA induced DA cell death. IL-1ra administered in the periphery has previously been shown to cross the blood brain barrier [36,37]. In support of this we show that IL-1ra administration reduces levels of the pro-inflammatory cytokines IFN-γ and TNF-α in the SN following three days of exposure. Cross regulation between IL-1β, TNF-α, and IFN-γ is not surprising considering the substantial overlap in intra-cellular/nuclear signaling they possess [61]. Sites of overlap include, phospholipid hydrolysis and protein tyrosine kinase phosphorylation in the cell and shared nuclear binding motifs, κB and NF-IL6 [62-65]. All three cytokines share the transcription factor Nf-κB, a key regulator of pro-inflammatory cytokine expression [66-68]. IL-1ra administration therefore may not have only attenuated cell loss through blockade of IL-1 signaling per se, but also these data suggest that regulation of other cytokines (TNF-α and IFN-γ) likely also contributed. Importantly, both IFN-γ and TNF-α are found to be increased in the human PD brain [69] and can be toxic to DA neurons when injected into the rat brain [57]. Interferon-γ was recently shown to be elevated in PD serum [70] and was implicated in MPTP induced DA neuron degeneration in mice [70]. In several reports, TNF-α signaling has been shown to be involved in the destruction of SN DA neurons in animal models of PD [31,70-73]. This report and others further point to the adverse role cytokines can play in DA neuron survival, while in contrast providing potential therapeutic targets.

IL-1ra delivery to the CNS has also been used successfully in a rodent stroke model where infarct size was significantly reduced [74]. In humans, IL-1ra administration (Anakinra, s.c., Amgen) is well tolerated and is approved for use in patients with rheumatoid arthritis. Further research into IL-1ra as a potential therapy in neurodegenerative disease is therefore warranted.

Inflammation produced by a single injection of a large dose of LPS into the periphery has recently been shown to produce inflammation in the brain and resulted in significant DA neuron loss in the SN at 7 months and progressively more so at 10 months in the absence of a second insult [75]. These data along with those reported here indicate that inflammation produced both locally and systemically have the potential to not only create DA neuron vulnerability, but also frank cell loss.

Our data fit with the threshold or "multiple hit" hypothesis of neurodegenerative disease and sporadic PD [9,10]. According to these hypotheses several risk factors of PD, including genetic predisposition, toxin exposure, aging, and other as of yet unknown factors interact to facilitate DA cell loss past the threshold of PD onset. Several reports support this hypothesis and reveal that disparate challenges drain multiple compensatory mechanisms leading to greater cell loss [11]. For example, we have previously shown that the combination of two otherwise non-toxic doses of NMDA and 3-nitropropionic acid (3-NP) results in striatal cell death [76]. Prenatal exposure to LPS produces increased DA cell loss following adult exposure to 6-OHDA [77]. Peng et al. recently showed that neonatal exposure to iron produces a greater loss of SN DA neurons combined with paraquat administration in adulthood (24 months old) [78]. The combination of genetic and environmental insults has also produced Parkinson's like pathology. It was found that a double-mutant human alpha-synuclein overexpressing mouse exposed to pesticides (maneb and paraquat) showed SN DA cell loss in greater magnitude than either single insult alone [79].

The present study suggests that preexisting neuroinflammation is a risk factor for the development of PD. This is supported clinically, by the finding that the relative risk of being diagnosed with PD is reduced by the use of NSIADs [12-14]. Interestingly, a clinical phenotype of PD emerged following the outbreak of the Spanish flu in 1918 [80] and while viral RNA is reported absent from brains of the deceased [81], it is conceivable that the massive immune response or "cytokine storm" [82] created by the virus initiated inflammation in the CNS. This has been shown in an experimental sepsis model, where LPS delivered systemically to mice leads to neuroinflammation and the eventual death of SN DA neurons with an apparent sparing of neighboring DA ventral tegmental neurons [75].

## Conclusion

In conclusion, these experiments demonstrate that mild inflammation caused by a bacterial endotoxin can increase the vulnerability of midbrain DA neurons to PD-like degeneration *in vivo*, and identify the specific changes in cytokine brain tissue levels associated with the increased risk of degeneration. Based on the tissue cytokine profile showing elevated IL-1β, systemic therapy using an IL-1 receptor antagonist provides direct evidence in animal models that the inflammation induced neuronal vulnerability can be counteracted. These data give new insights into how low-grade inflammation may trigger the onset of PD and other neurodegenerative diseases and the specific therapeutic avenues to limit such pathogenesis.

## Competing interests

The author(s) declare that they have no competing interests.

## Authors' contributions

JK designed the studies, performed stereotaxic surgery, performed cell counting stereology, prepared tissue for ELISA, and wrote and prepared the manuscript. CRN performed stereotaxic surgery, was involved in experimental design, and helped write the manuscript. PM performed stereotaxic surgery, was involved in experimental design, and reviewed the manuscript. OI conceived the idea for the study and designed the experiments, reviewed the data, and wrote the manuscript. All authors read and approved the final manuscript.
